# Metallothioneins regulate ATP7A trafficking and control cell viability during copper deficiency and excess

**DOI:** 10.1038/s41598-020-64521-3

**Published:** 2020-05-12

**Authors:** Nikita Gudekar, Vinit Shanbhag, Yanfang Wang, Martina Ralle, Gary A. Weisman, Michael J. Petris

**Affiliations:** 1 The Genetics Area Program, University of Missouri, Columbia, MO 65211 USA; 2 The Christopher S. Bond Life Sciences Center, University of Missouri, Columbia, MO 65211 USA; 3 The Department of Biochemistry, University of Missouri, Columbia, MO 65211 USA; 4 The Department of Nutrition and Exercise Physiology, University of Missouri, Columbia, MO 65211 USA; 5 The Department of Molecular and Medical Genetics, Oregon Health & Science University, Portland, Oregon, 97239 USA; 6 Present Address: State Key Laboratory of Animal Nutrition, Institute of Animal Science, Chinese Academy of Agricultural Sciences, Beijing, 100193 People’s Republic of China

**Keywords:** Biochemistry, Metals, Metabolic disorders, Cell biology

## Abstract

Copper (Cu) is an essential, yet potentially toxic nutrient, as illustrated by inherited diseases of copper deficiency and excess. Elevated expression of the ATP7A Cu exporter is known to confer copper tolerance, however, the contribution of metal-binding metallothioneins is less clear. In this study, we investigated the relative contributions of ATP7A and the metallothioneins MT-I and MT-II to cell viability under conditions of Cu excess or deficiency. Although the loss of ATP7A increased sensitivity to low Cu concentrations, the absence of MTs did not significantly affect Cu tolerance. However, the absence of all three proteins caused a synthetic lethal phenotype due to extreme Cu sensitivity, indicating that MTs are critical for Cu tolerance only in the absence of ATP7A. A lack of MTs resulted in the trafficking of ATP7A from the *trans*-Golgi complex in a Cu-dependent manner, suggesting that MTs regulate the delivery of Cu to ATP7A. Under Cu deficiency conditions, the absence of MTs and / or ATP7A enhanced cell proliferation compared to wild type cells, suggesting that these proteins compete with essential Cu-dependent pathways when Cu is scarce. These studies reveal new roles for ATP7A and metallothioneins under both Cu deficiency and excess.

## Introduction

Copper (Cu) is an essential enzymatic cofactor in organisms across all phyla^1,2^. The ability of Cu to cycle between Cu^1+^ and Cu^2+^ allows cuproenzymes to catalyze redox reactions in many different areas of metabolism^3^. In eukaryotic cells, Cu is imported to the cytoplasm by members of the SLC31 family of high-affinity Cu permeases^4^. This process is thought to be facilitated by Cu binding to glutathione in the cytoplasm^5^. Glutathione-bound Cu is exchangeable with metallochaperones^6,7^, which are small cytoplasmic proteins that deliver Cu in a unidirectional manner to specific cuproenzymes or to Cu-transporting P-type ATPases located in the Golgi. This targeted delivery of Cu is thought to be facilitated by an increasing affinity gradient down successive Cu carriers as well as metal-dependent interactions between metallochaperones and target proteins^3,6,8,9^.

The same redox property that makes Cu indispensable as an enzymatic cofactor is also responsible for its toxicity when cytoplasmic concentrations become elevated. In its free ionic state, Cu can catalyze the formation of hydroxyl radicals, displace other metals from enzymes and interfere with protein folding^10,11^. Accordingly, mechanisms of Cu homeostasis have evolved to prevent free Cu ions from accumulating within the cytoplasm. Virtually all organisms are predicted to contain at least one P-type ATPase that exports Cu from the cytoplasm. In bacteria and mammalian cells, mutations that disrupt Cu-ATPases are known to cause hypersensitivity to Cu, whereas overexpression of these transporters results in Cu resistance^11,12^. Mammals possess two Cu-transporting ATPases, ATP7A and ATP7B. ATP7A is expressed ubiquitously, whereas ATP7B is restricted to specific tissues with highest levels in hepatocytes^13^. At the cellular level, both transporters are known to reside within the trans-Golgi network (TGN)^14–16^. At this location, ATP7A and ATP7B transport Cu to nascent cuproenzymes within the secretory pathway^16,17^. Previous studies using cultured cell lines have demonstrated that elevated Cu concentrations stimulate the trafficking of ATP7A and ATP7B to the plasma membrane or to endolysosomal compartments, respectively^15,18^. This trafficking response is a homeostatic mechanism that prevents the overaccumulation of potentially toxic Cu by facilitating Cu export across the plasma membrane or compartmentalization within post-Golgi vesicles/lysosomes.

Another conserved mechanism of Cu tolerance is metal sequestration by metallothioneins (MTs). These small cysteine-rich proteins have a high affinity for heavy metals including Cu, Cd, Zn and Hg^19^. In single-cell eukaryotes, MTs predominantly bind Cu and are required for tolerance to this metal^20^. Mammals possess at least four distinct MT genes (MT-I through MT-IV), with MT-I and MT-II being the major isoforms that are ubiquitously expressed throughout development^21^. MT-III and MT-IV are minor isoforms that are predominantly expressed in neurons and glia or in stratified epithelia, respectively^22,23^. Both MT-I and MT-II are transcriptionally induced by heavy metals via the MTF-1 transcription factor in a Zn-dependent manner^24^. MTs have been shown to bind Zn with high affinity, which can be readily exchanged for Cu^25^. Cultured cells lacking MTs exhibit higher sensitivities to Cu, suggesting that MTs can protect against Cu *in vitro*^26^. This concept is further supported by the finding that forced expression of MTs increases Cu tolerance in cultured cells^27^. However, mice lacking both MT-I and MT-II do not exhibit an increased sensitivity to toxic levels of Cu, suggesting that MTs alone do not play a major role in Cu tolerance *in vivo*^28^. The extent to which MTs are protective against Cu may be dependent on the expression of ATP7A, as the deletion of MT-I and MT-II causes embryonic lethality in mice lacking a functional ATP7A transporter^29^.

In this study, we generated isogenic cell lines to examine the importance of ATP7A, MT-I and MT-II (MTs) to Cu homeostasis. Whereas the loss of MTs or ATP7A reduced cell proliferation in media containing elevated Cu concentrations, deletion of all three genes resulted in a loss of cell viability due to extreme sensitivity to endogenous Cu in the medium. Additionally, we found that the absence of MTs stimulated the trafficking of ATP7A from the *trans*-Golgi network to cytoplasmic vesicles, suggesting that MTs regulate the availability of Cu for ATP7A trafficking. In contrast to wild type cells, cells lacking MTs or ATP7A were viable under conditions of Cu scarcity, indicating that MTs and ATP7A restrict the flow of Cu to essential Cu-dependent metabolic pathways. Our results reveal new roles for ATP7A and metallothioneins under conditions of Cu deficiency and excess.

## Results

### Generation of cell lines lacking Atp7a, MtI and MtII genes

**Figure 1:**
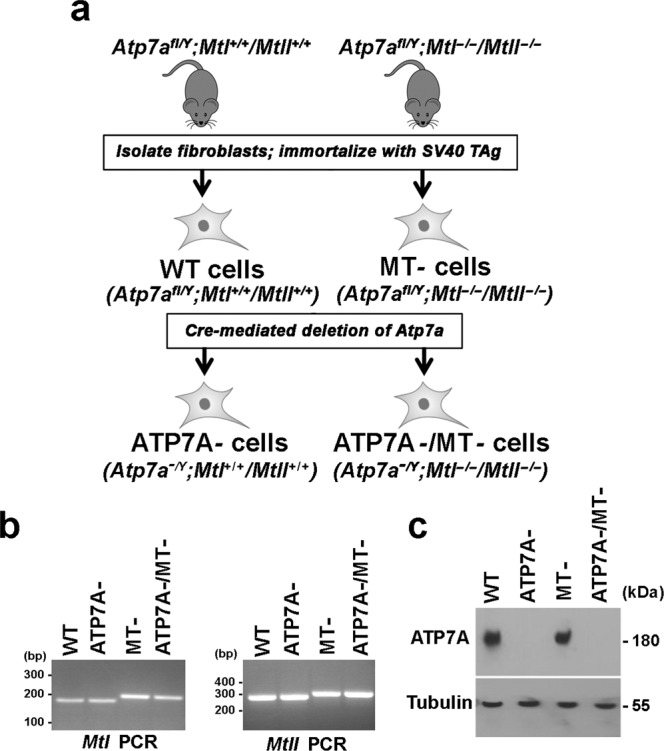
Derivation and characterization of cell lines lacking *Atp7a*, *MtI* and *MtII* genes. (**a**) Primary fibroblasts were isolated from the lungs of *Atp7a*^*fl/Y*^*;MtI*^*+/+*^*/MtII*^*+/+*^ and *Atp7a*^*fl/Y*^*;MtI*^*−/−*^*/MtII*^*−/−*^ mice and then immortalized by transfection with a plasmid expressing the SV40 large T antigen (SV40 Tag) resulting in WT and MT- cells, respectively. An adenoviral vector encoding CRE recombinase was used to delete *Atp7a* in WT and MT- cells to obtain ATP7A- and ATP7A-/MT- cells, respectively. (**b**) PCR analysis of genomic DNA was used to confirm deletion of *MtI* and *MtII* genes in both the MT- and ATP7A-/MT- cell lines. Expected PCR product sizes: *MtI* gene (WT = 161 bp; knockout = 176 bp); *MtII* gene (WT = 282 bp; knockout = 299 bp). (**c**) Immunoblot analysis was used to confirm the loss of ATP7A protein in both ATP7A- and ATP7A-/MT- cell lines. Tubulin was detected as a loading control. Images of full-length gels and immunoblots are provided in the supplementary data.

To investigate the requirement for ATP7A and MTs in copper homeostasis, we used two different mouse strains to derive immortalized cell lines with deletions in the *Atp7a* gene with or without deletion of both *MtI* and *MtII* genes (Fig. 1a). Floxed *Atp7a* male mice (Atp7a^fl/Y^*;MtI*^*+/+*^*;MtII*^*+/+*^)^30^ were crossed with female mice carrying deletions of both *MtI* and *MtII* genes^31^ (Supplementary Fig. S1). A cross between heterozygous F1 males and females was then used to generate Atp7a^fl/Y^*;MtI*^*−/−*^*;MtII*^*−/−*^ mice (Supplementary Fig. S1). Fibroblasts were isolated from the lungs of both Atp7a^fl/Y^*;MtI*^*+/+*^*;MtII*^*+/+*^ and Atp7a^fl/Y^*;MtI*^*−/−*^*;MtII*^*−/−*^ mice and subsequently immortalized using a plasmid encoding the SV40 large T antigen to obtain WT and MT- cell lines (Fig. 1a). To delete the *Atp7a* gene, both cell lines were infected with an adenovirus expressing Cre recombinase (Ad-Cre) to generate ATP7A- cells (Atp7a^−/Y^*;MtI*^*+/+*^*;MtII*^*+/+*^) and ATP7A-/MT- cells (*Atp7a*^*−/Y*^*;MtI*^*−/−*^*;MtII*^*−/−*^) (Fig. 1a). Surprisingly, the introduction of Ad-Cre virus into MT- cells to delete the *Atp7a* gene resulted in a complete loss of cell viability in basal medium, suggesting that the combined loss of ATP7A and both MTs results in lethality (Supplementary Fig. S2).

Although the endogenous Cu concentrations in basal medium are quite low (1.7 µM), we considered the possibility that the removal of ATP7A from MT- cells might cause extreme sensitivity to Cu, thus preventing their propagation in basal medium. To test this possibility, we deleted the *Atp7a* gene in MT- cells using Ad-Cre virus as before, but this time recovered the cells in basal medium containing the extracellular Cu chelator, bathocuproine disulfonate (BCS). This permitted the robust growth of ATP7A-/MT- clones, which could be propagated indefinitely in BCS-containing medium (Supplementary Fig. S2). PCR analysis of genomic DNA confirmed the *MtI* and *MtII* genotypes of each cell line (Fig. 1b). The presence or absence of the ATP7A protein was confirmed by immunoblot analysis of each cell line, with tubulin serving as a loading control (Fig. 1c). These findings suggest that loss of ATP7A and MTs causes a synthetic lethal genetic interaction due to extreme Cu sensitivity.

### Characterization of the ATP7A-/MT- cells

**Figure 2:**
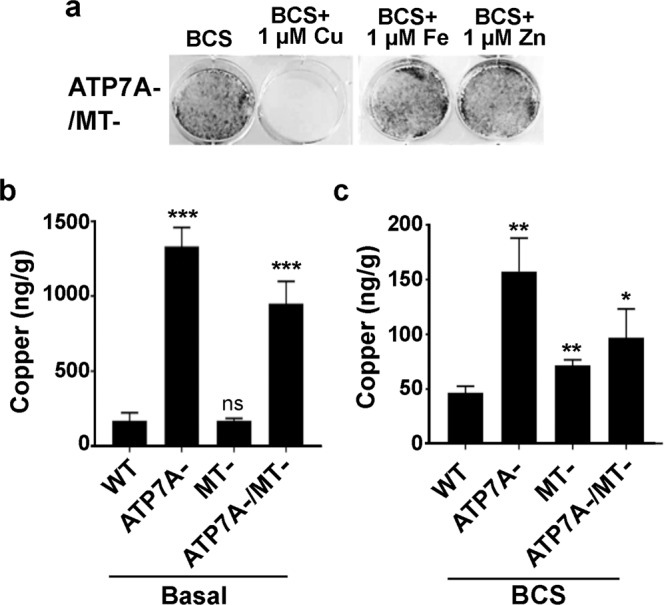
Disruption of *Atp7a* and *MtI/II* results in a loss of cell viability that is suppressed by Cu chelation. (**a**) The rescue of ATP7A-/MT- cells by the Cu chelator BCS is suppressed by copper, but not zinc or iron. ATP7A-/MT- cells were grown for 5 days in basal media containing 1 µM BCS with or without equimolar concentrations of CuCl_2_, ZnCl_2_ or FeCl_2_. Cell survival was then determined using the Crystal Violet assay and imaged. (**b,c**) Cu concentrations in each cell line were determined by ICP-MS. Cells were grown for 2 days in medium containing 50 µM of the copper chelator BCS and then exposed for 24 h to either basal media or media supplemented with 50 µM BCS (mean ± SEM; *p < 0.05; **p < 0.01; ***p < 0.001; ns = not significant).

To test whether the ability of BCS to rescue ATP7A-/MT- cells in basal medium was in fact attributable to Cu chelation, we tested whether the addition of equimolar Cu, Fe or Zn to the BCS-containing media could suppress the rescue of these cells. Of these metals, only Cu was found to prevent the rescue of ATP7A-/MT- cells by BCS (Fig. 2a), thus confirming that the ATP7A-/MT- cells are inviable in basal medium due to Cu toxicity. Next, we measured the total Cu concentrations in each cell line grown in either basal medium or BCS-containing medium using inductively coupled plasma mass spectrometry (ICP-MS). Since Cu toxicity in ATP7A-/MT- cells requires exposure to basal medium for at least 96 h, Cu measurements were performed on cells initially grown for two days in BCS-containing medium and then exposed to either basal medium or BCS-containing medium for a further 24 h. Compared to WT cells, the intracellular Cu concentrations were significantly elevated in both the ATP7A- and ATP7A-/MT- cells exposed to basal medium (Fig. 2b). In contrast, there was no difference in Cu accumulation between WT and MT- cells exposed to basal medium (Fig. 2b). As expected, BCS reduced the accumulation of Cu in all cell lines compared to basal medium, however, each mutant cell line still contained significantly more Cu than WT cells under these conditions (Fig. 2c). Compared to WT cells, the mutant cell lines contained more Fe and Zn under basal and BCS conditions, however, these increases did not reach significance for every mutant (Supplementary Fig. S3).

**Figure 3:**
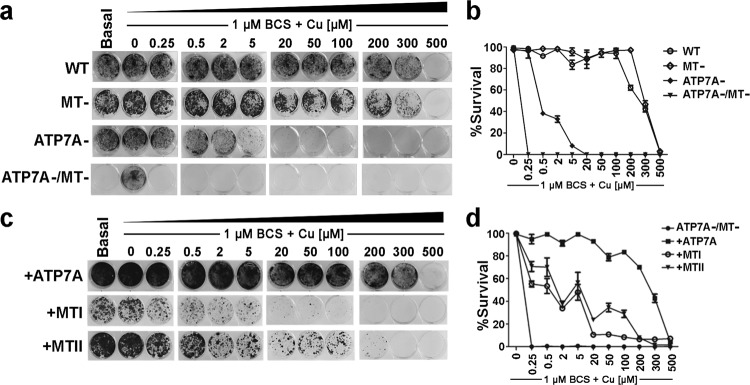
Relative contributions of ATP7A and metallothioneins to Cu tolerance. (**a**) Cu sensitivity of WT, ATP7A-, MT- and ATP7A-/MT- cells. For each cell line, 10^3^ cells/well were seeded in 6-well plates containing basal medium, or basal medium containing 1 µM BCS with or without the indicated concentrations of CuCl_2_. After 6 days, cell survival was determined using the Crystal Violet assay and imaged. (**b**) Quantification of Crystal Violet staining. Data are expressed as percent cell survival for each cell line normalized against its growth in BCS (mean ± SEM). (**c**) Complementation of ATP7A-/MT- cells with plasmids encoding human cDNAs for *ATP7A* ( + ATP7A), *MTI* ( + MTI) or *MTII* ( + MTII). Cells were transfected with each plasmid and then selected in basal medium supplemented with 1 µM CuCl_2_. Equal numbers of surviving cells (10^3^ cells/well) were then seeded into 6-well plates containing basal medium, 1 µM BCS or 1 µM BCS plus the indicated concentrations of Cu. After 6 days, cell survival was determined using the Crystal Violet assay and imaged. (**d**) Quantification of Crystal Violet staining. Data are expressed as percent cell survival for each cell line normalized against its growth in BCS (mean ± SEM).

To further characterize the contribution of ATP7A and MT to Cu tolerance, we assessed the viability of each cell line exposed to increasing Cu concentrations added to the media. Cells were cultured in either basal medium alone or containing 1 µM BCS with or without various Cu concentrations. After 6 days, cell survival was assayed. The results showed that the MT- cells were only marginally more sensitive to Cu as compared to WT cells (Fig. 3a,b). In contrast, the survival of ATP7A- cells was markedly decreased compared to both WT and MT- cells (Fig. 3a,b). The ATP7A-/MT- cells were completely inviable in as little as 0.25 µM Cu added to the BCS-containing medium (Fig. 3a,b). Additional studies found that approximately 0.05 µM Cu added to medium containing 1 µM BCS was sufficient to reduce the viability of ATP7A-/MT- cells by 50% (Supplementary Fig. S4).

Previous studies suggest that the majority of cytoplasmic Cu is bound to glutathione, a highly abundant thiol-containing tripeptide that helps maintain the reducing potential of the cytosol^32,33^. Excess Cu concentrations are known to disrupt glutathione homeostasis by decreasing the ratio of reduced glutathione (GSH) to oxidized glutathione (GSSG)^34^. Thus, we tested whether glutathione homeostasis might be disrupted by the loss of ATP7A and/or MT in cells grown in basal medium. Compared to WT cells, total GSH levels were significantly increased in both the ATP7A- and ATP7A-/MT- cell lines (Supplementary Fig. S5a). In the MT- cells there was a small but significant decrease in total GSH levels (Supplementary Fig. S5a). Notably, in each mutant cell line the ratio of GSH to GSSG was significantly decreased compared to WT cells (Supplementary Fig. S5b). These data indicate that the loss of ATP7A and/or MT significantly disrupts glutathione homeostasis.

Next, we performed complementation analyses by transfecting ATP7A-/MT- cells with expression constructs for human ATP7A, MTI or MTII, followed by selection in basal media supplemented with 1 µM Cu. Surviving cells were then expanded in basal media for further analysis. The forced expression of either MTI or MTII was found to complement the growth of ATP7A-/MT- cells, albeit at a lower magnitude compared to force expression of ATP7A (Fig. 3c,d). Taken together, these findings suggest that ATP7A is the primary mediator of cellular Cu tolerance and that MTI and MTII provide secondary roles that become critical in the absence of ATP7A.

### Suppressor mutations in ATP7A-/MT- cells increase ATP7B expression and confer copper tolerance

**Figure 4:**
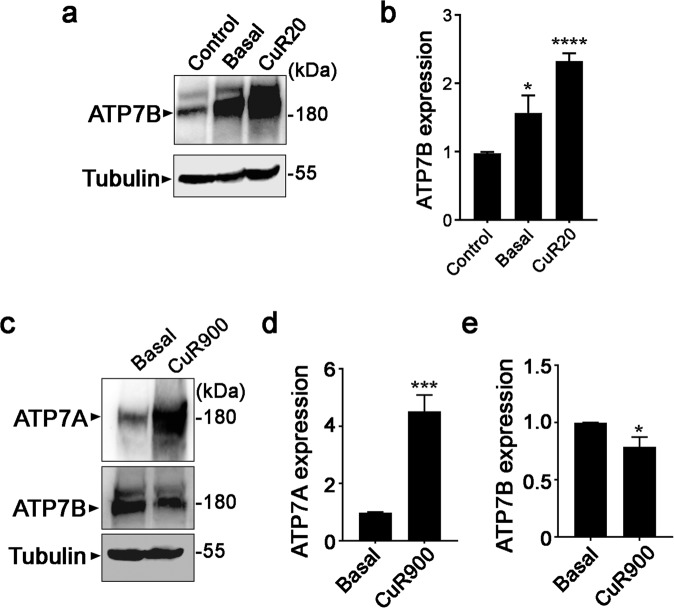
Selection for Cu tolerance in the absence of ATP7A increases ATP7B expression. (**a**) Immunoblot detection of ATP7B in ATP7A-/MT- cells that were selected for survival in either basal media (Basal) or media containing 20 µM Cu (CuR20). The control sample is ATP7A-/MT- cells propagated in medium containing 50 µM BCS. Each cell line was grown for 16 h in basal media prior to immunoblot analysis. Tubulin was detected as a loading control. (**b**) Densitometry analysis of ATP7B protein levels normalized against control samples (mean ± SEM; *p < 0.05; ****p < 0.0001). Data were calculated from at least 3 independent experiments. (**c**) Immunoblot detection of ATP7A and ATP7B proteins in parental ATP7A + /MT- cells (Basal) or ATP7A + /MT- cells that were selected in media containing 900 µM Cu (CuR900). Both cell lines were grown for 16 h in basal media prior to immunoblot analysis. Tubulin was detected as a loading control. (**d,e**) Densitometry analysis of ATP7A and ATP7B protein levels normalized against Basal samples (mean ± SEM; *p < 0.05; ***p < 0.001). Data were calculated from at least 3 independent experiments. Images of full-length immunoblots are provided in the supplementary data.

Over the course of our studies, we observed rare instances of Cu-resistant ATP7A-/MT- cells that spontaneously formed colonies in basal media. Because ATP7A-/MT- cells contain large deletions within the *Atp7a* and *MtI/II* genes, we hypothesized that these suppressor mutations were not the result of genetic reversions, but rather induced alternative pathways of Cu handling. ATP7B is a Cu-transporting P-type ATPase that shares approximately 57% identity with ATP7A, and has been shown to functionally replace ATP7A in cultured fibroblasts^35,36^. Thus, we screened the Cu-resistant ATP7A-/MT- cells for possible increases in ATP7B expression. The ATP7A-/MT- colonies were pooled and expanded in basal medium. A subset was further selected in basal media in which Cu concentrations were gradually increased to 20 µM over the course of 50 days while passaging cells every 2–3 days. Compared to the parental ATP7A-/MT- cells, the ATP7A-/MT- cells that were resistant to either basal medium or 20 µM Cu expressed higher levels of the ATP7B protein (Fig. 4a,b). Quantitative PCR analysis demonstrated that the abundance of *Atp7b* mRNA was also increased in these Cu-resistant cell lines (Supplementary Fig. S6a). These results suggest that spontaneous overexpression of ATP7B is a mechanism for restoring Cu tolerance in ATP7A-/MT- cells.

In a separate experiment, we tested whether Cu selection would result in increased ATP7B expression in cells containing a functional ATP7A. Therefore, we passaged ATP7A + /MT- cells in media containing step-wise increases in Cu concentrations until resistance to 900 µM Cu was achieved. The Cu-resistant ATP7A+/MT- cells exhibited increased expression of both ATP7A protein (Fig. 4c,d) and *Atp7a* mRNA (Supplementary Fig. S6b) compared to the parental cells grown in basal medium. However, there was no increase in ATP7B protein in these Cu-resistant cells, and indeed for reasons that are unclear there was a small but significant decrease (Fig. 4c,e), which was also observed at the mRNA level (Supplementary Fig. S6c). These data suggest that elevated ATP7A expression is the major driver of Cu tolerance and that the selection for elevated ATP7B expression occurs in the absence of ATP7A.

### Metallothioneins negatively regulate Cu-stimulated trafficking of ATP7A from the Golgi

**Figure 5:**
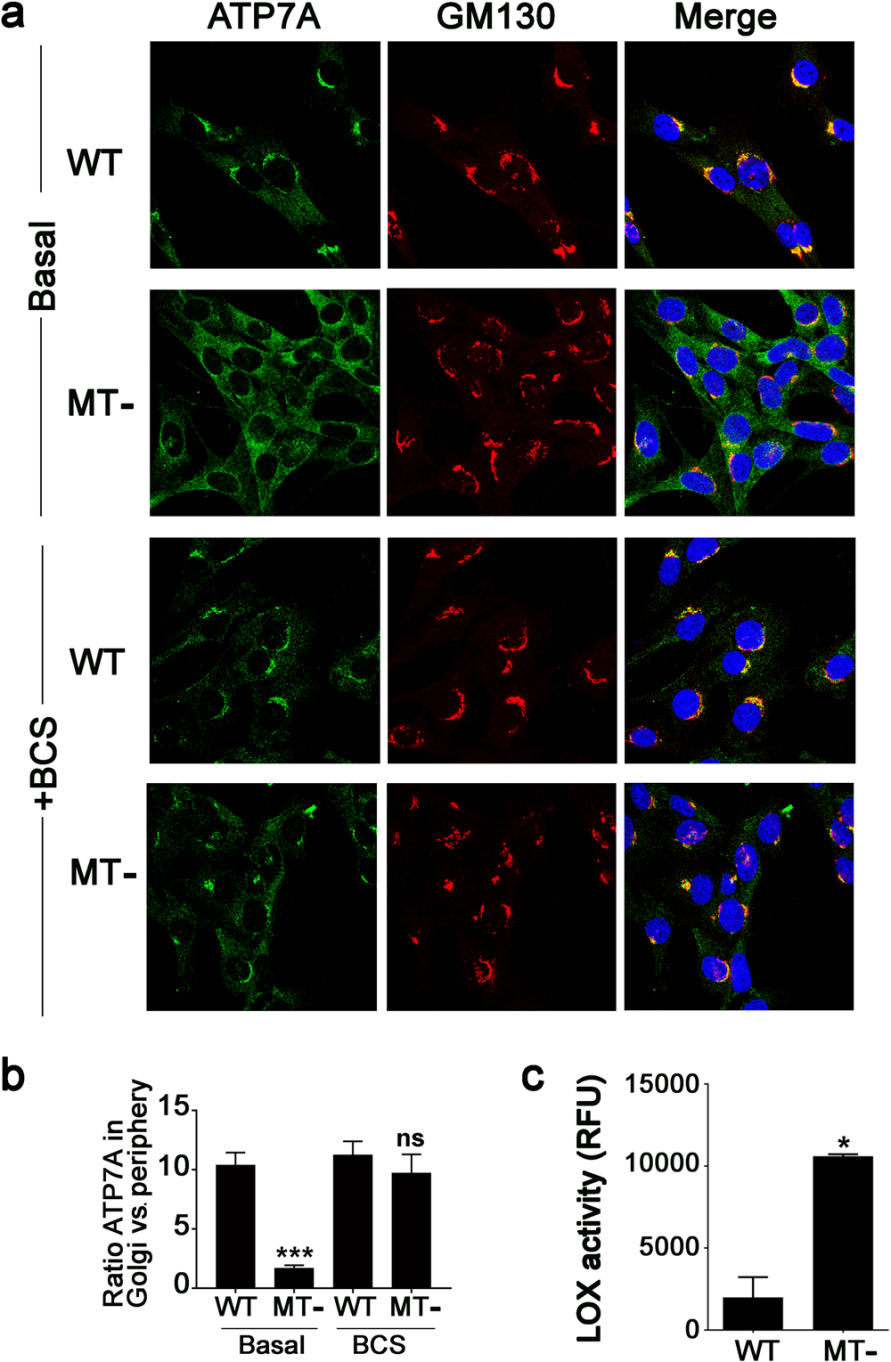
Metallothionein regulates Cu-stimulated ATP7A trafficking and function. (**a**) Immunofluorescence microscopy was performed to detect endogenous ATP7A (green) in WT and MT- cells cultured for 24 h in basal medium alone or basal medium supplemented with 50 µM BCS. Antibodies against GM130 were used to label the Golgi complex (red) and DAPI was used to stain the nuclei (blue). (**b**) The ATP7A staining intensity in the Golgi region was expressed relative to the ATP7A staining intensity within the non-Golgi region for each cell line. Values were calculated using ImageJ software (mean ± SEM; ***p < 0.001; ns = not significant; n = 24 cells for WT and MT- cells for each condition). (**c**) Lysyl oxidase (LOX) activity was measured in the media collected from WT and MT- cells cultured for 48 h (mean ± SEM; *p < 0.05).

Previous studies demonstrate that ATP7A confers Cu tolerance, which is coupled with the Cu-stimulated trafficking of ATP7A from the Golgi to vesicular compartments or the plasma membrane^15^. To explore the potential impact of MTs on this process, we used quantitative immunofluorescence microscopy to investigate the distribution of endogenous ATP7A protein in both WT and MT- cells. In WT cells grown in basal medium the ATP7A protein was predominantly localized in a perinuclear region, which overlapped with the Golgi marker protein, GM130 (Fig. 5a,b). However, in MT- cells cultured in basal medium, there was a significantly greater fraction of the ATP7A protein located within cytoplasmic vesicles that extended outside of the perinuclear region to the cell periphery (Fig. 5a,b). The addition of BCS to the medium restored ATP7A to the perinuclear region in MT- cells, which overlapped with GM130 (Fig. 5a,b). These findings suggest that in the absence of MTs, there is more Cu available to stimulate trafficking of ATP7A from the Golgi. Consistent with these findings, the activity of the secreted Cu-dependent enzyme lysyl oxidase (LOX), which receives Cu from ATP7A^37^, was found to be elevated in the media of MT- cells compared to WT cells (Fig. 5c). Taken together, these results suggest that MTs lower the availability of Cu for ATP7A trafficking and transport.

### ATP7A and MTs are determinants of cell viability during copper starvation

**Figure 6:**
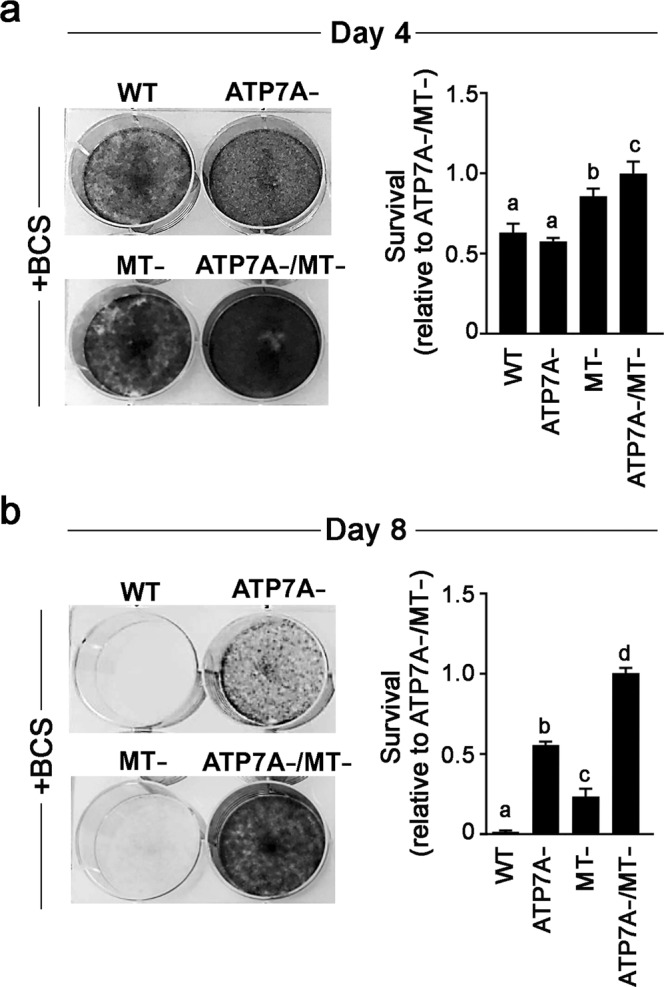
ATP7A and MTs decrease cell viability during Cu starvation. (**a**) WT, ATP7A-, MT- and ATP7A-/MT- cells were seeded into 6-well plates (2 ×10^4^ cells/well) and cultured in medium supplemented with 50 µM BCS. After 4 days, cells were stained with Crystal Violet and imaged. (**b**) A second batch of identically treated cells was passaged for an additional 4 days in BCS-containing medium and then stained with Crystal Violet (*i.e*., after 8 days of Cu chelation). Crystal Violet staining in both (**a**) and (**b**) was quantified as a measure of cell survival. Values were normalized against the survival of the ATP7A-/MT- cell line in BCS at Day 4 and Day 8 (mean ± SEM). Different letters indicate values that are significantly different from each other. Note that the loss of ATP7A or MTI/II enhances the ability of cells to propagate in the presence of the BCS copper chelator.

The finding that MTs restrict Cu delivery to ATP7A raises the question of whether MTs also control the availability of Cu for processes that are necessary for cell viability during Cu deficiency. Thus, we investigated whether the presence of MTs or ATP7A affects cell propagation under conditions of Cu scarcity. WT, MT-, ATP7A- and ATP7A-/MT- cells were seeded at low densities in media containing 50 µM of the extracellular Cu chelator BCS. After 4 days, all cells remained viable (Fig. 6a). Passaging of these cells for a further 4 days in the continued presence of BCS prevented the propagation of WT cells (Fig. 6b). In contrast, MT-, ATP7A- and ATP7A-/MT- cells continued to propagate in BCS-containing media, with the most robust growth observed for both ATP7A- and ATP7A-/MT- cells (Fig. 6b). These findings suggest that during Cu deficiency, ATP7A and to a lesser extent MTs restrict the availability of Cu for essential metabolic functions.

## Discussion

Because Cu is highly toxic when present in the free ionic form, the maintenance of cellular Cu homeostasis must be strictly controlled. The purpose of the current study was to investigate the relative contributions of ATP7A, MTI and MTII to Cu homeostasis. Consistent with the role of ATP7A in Cu export, we found that ATP7A- cells accumulated higher levels of Cu compared to either WT or MT- cells, and were more sensitive to this metal (Figs. 2 and 3). In contrast, the MT- cells were only marginally more sensitive to Cu at very high concentrations (>100 µM) (Fig. 3). These findings indicate that ATP7A is the principal driver of Cu tolerance and suggest that the contribution of MTs is not essential in cells with a functional ATP7A Cu transporter. Although both the ATP7A- and the ATP7A-/MT- cells were found to hyperaccumulate Cu to significantly greater levels than either WT or MT- cells when grown in basal media (Fig. 2), only the ATP7A-/MT- cells were inviable under these conditions (Fig. 3). From these results we conclude that MTs are necessary to protect cells against Cu overload caused by the absence of ATP7A. Compared to WT cells, concentrations of Fe and Zn were found to be elevated in cells lacking either ATP7A and/or MT, although these increases did not reach significance for all mutants (Supplementary Fig. S3). Since excess Cu is known to out-compete Fe and Zn for metalloenzymes^10,24,38,39^, it is possible that the accumulation of Fe and Zn in these mutant cells is attributable to Cu-mediated inhibition of export pathways for these metals.

The majority of cytoplasmic Cu is bound to glutathione, an antioxidant tripeptide present at millimolar concentrations within eukaryotic cells^32,33^. Previous studies have shown that ATP7A-null fibroblasts are sensitive to glutathione depletion, suggesting that glutathione can protect against Cu hyperaccumulation in the absence of ATP7A^40^. Consistent with these studies, we found that the abundance and oxidation of glutathione were markedly increased in cells lacking ATP7A and/or MTs (Supplementary Fig. S5). However, since ATP7A-/MT- cells are inviable, this indicates that glutathione is unable to compensate for the loss of ATP7A and MTs. The inviability of ATP7A-/MT- cells is not because Cu concentrations exceed the binding capacity of glutathione, since GSH concentrations are more abundant than Cu by several orders of magnitude. Rather, we postulate that in the absence of the high affinity Cu sinks provided by ATP7A and MTs, glutathione-bound Cu is too labile to prevent deleterious interactions of Cu with intracellular targets. This model is consistent with the prevailing concept of glutathione as a low affinity, high capacity Cu buffer that enables facile Cu exchange with high affinity targets such as metallochaperones, metallothionein and copper-dependent enzymes^6,41^.

Previous studies have shown that elevated cytoplasmic Cu concentrations stimulate ATP7A trafficking from the Golgi complex into vesicles that ultimately fuse with the plasma membrane^15^. The initiation of Cu-stimulated ATP7A trafficking is dependent on its transport function as well as Cu delivery via the ATOX1 metallochaperone^42,43^. It was therefore notable that the Cu-dependent trafficking of ATP7A from the *trans*-Golgi and its transport activity (*i.e*., LOX secretion) were both increased in MT- cells relative to WT cells in basal medium (Fig. 5). This effect was not due to increased concentrations of total Cu in the MT- cells, which were identical to WT levels under basal growth conditions (Fig. 2b). However, the finding that BCS restored ATP7A localization to the Golgi indicates that ATP7A trafficking in the absence of MT was caused by increased Cu availability. These findings suggest that when endogenous Cu is low, MTs limit Cu availability to ATP7A, thus ensuring that ATP7A trafficking is not inappropriately activated until Cu concentrations become elevated.

Our studies with ATP7A-/MT- cells prompted us to test whether ATP7A and MTs influence cell viability under conditions of Cu scarcity. In media containing the extracellular Cu chelator, BCS, wild type cells failed to propagate after two passages whereas the ATP7A-, MT-, and ATP7A-/MT- cells continued to propagate under these conditions (Fig. 6). These data suggest that when extracellular Cu is limited, intracellular Cu is withheld from essential metabolic processes either by direct sequestration by MTs, or by removal from the cytoplasm by ATP7A. Our results are consistent with *in vitro* studies demonstrating that apo-MT-II is able to rapidly extract copper ions from metallochaperones^6^, and suggest that MTs act as irreversible sinks for Cu rather than serving as Cu storage pools that can be drawn upon in the absence of Cu import. Similarly, our findings suggest that ATP7A-dependent transport of Cu into the Golgi or post-Golgi vesicles is an irreversible process, rather than a means of storing Cu that can be released into the cytoplasm during Cu deficiency. Such a model may appear counter intuitive, since Cu tolerance mechanisms are expected to be non-functional during copper scarcity so that any available metal ion can be used for metabolic purposes. However, previous studies in *Saccharomyces cerevisiae* have shown that the vacuolar zinc importer, ZRC1, is induced by zinc deficiency to protect cells against a rapid influx of zinc when concentrations of this metal return to normal^44^. Thus, ATP7A and MTs may similarly function to safeguard against Cu toxicity when levels of this metal are restored. These findings have implications for understanding copper metabolism during pathological Cu deficiency in humans. For example, excess zinc intake due to accidental overdose is a risk factor for potentially lethal Cu deficiency^45–47^. Because zinc induces the expression of MTs^24^, the results of our study highlight a potential mechanism whereby excess MT limits Cu availability for essential processes.

The extreme sensitivity of ATP7A-/MT- cells to Cu raises the possibility of using these cells to identify novel Cu tolerance genes. Support for this concept was our finding that spontaneous overexpression of ATP7B restores Cu tolerance in ATP7A-/MT- cells (Fig. 4). Virtually all eukaryotic metallochaperones and Cu transporters identified to date have been cloned by complementation of mutants in the baker’s yeast, *Saccharomyces cerevisiae*. It is therefore likely that certain Cu homeostasis genes in complex multicellular organisms are yet to be identified. The ATP7A-/MT- cells described in our study may be useful for identifying such genes.

### Experimental procedures

#### Animals

All animal experimentation methods were carried out in accordance with the regulations set forth by the National Institutes of Health Office of Laboratory Animal Welfare, and with the approval of the University of Missouri’s Animal Care and Use Committee. *Atp7a* floxed (*Atp7a*^*fl/Y*^) mice on the C57BL/6 background were generated in our laboratory, as previously described^30^. *MtI* and *MtII* double knockout mice^31^ on the 129/SvJ background were obtained from the Jackson Laboratories (strain 002211). Standard breeding procedures were used to obtain mice with the genotypes *Atp7a*^*fl/Y*^*; MtI*^*+/+*^*/MtII*^*+/+*^ (WT) and *Atp7a*^*fl/Y*^*; MtI*^*−/−*^*/MtII*^*−/−*^ (MT-).

#### Cell lines

Primary fibroblasts were obtained from WT and MT- mice by mincing freshly isolated lungs in Roswell Park Memorial Institute (RPMI) medium containing 4 mg/ml collagenase D and 2.5 mM CaCl_2_, digesting for 1 h at 37 °C, and then culturing at 37 °C in 5% CO_2_ in RPMI media containing 10% (v/v) FBS, 2 mM glutamine and 100 U/ml penicillin-streptomycin. Primary WT and MT- fibroblasts were immortalized by transfection with the pSV3 plasmid expressing the SV40 large T antigen^48^ using Lipofectamine 2000 (Invitrogen). Colonies were isolated and expanded in Dulbecco’s Modified Eagle Medium (DMEM) containing 10% (v/v) FBS, 4.5 g/L glucose, 2 mM glutamine and 100 U/ml penicillin-streptomycin (referred throughout as basal medium). To obtain ATP7A- and ATP7A-/MT- cell lines, the floxed *Atp7a* allele was disrupted in WT and MT- cell lines, respectively, using an adenoviral vector expressing GFP-tagged CRE recombinase (Ad-Cre-GFP 1700; Vector Biolabs). Individual clones were screened by immunofluorescence for the loss of ATP7A and then expanded. PCR was used to confirm the genotype of *MtI* and *MtII* in all cell lines, as described (https://www.jax.org/strain/002211). ATP7A-/MT- cells were propagated in basal media containing 50 µM of the Cu chelator bathocuproine disulfonate (BCS).

#### Plasmids

Expression plasmids for human *MTI* and *MTII* genes were purchased from Origene (RC205942 and RC202748, respectively). Human *ATP7A* plasmid pCMB344 was described previously^42^. ATP7A-/MT- cells were complemented by transfection with the above plasmids using Lipofectamine 2000, followed by selection in basal media with 1 µM CuCl_2_.

#### Cell viability analysis

Cells were seeded into 6-well trays (10^3^ cells/well) into basal media containing either BCS or CuCl_2_, as indicated in the figures. Cell survival was measured using a Crystal Violet assay, as described previously^49^.

#### Immunoblotting and immunofluorescence microscopy

Immunoblot analysis^50^ and immunofluorescence microscopy^51^ were performed as described. Antibodies used were anti-ATP7A^30^, anti-tubulin (Sigma, T8328), anti-GM130 (BD Biosciences; 610822), horse radish peroxidase-conjugated anti-rabbit IgG (Santa Cruz; SC2357), anti-mouse IgG (ThermoFisher; 31430), and Alexa Fluor 488-conjugated anti-rabbit (ThermoFisher; A11034) and Alexa Fluor 594-conjugated anti-mouse IgG (ThermoFisher; A11032). Rabbit polyclonal anti-ATP7B antibodies were produced commercially by Primm Biotech against a recombinant murine ATP7B protein comprising amino acids 316–491 and 1373–1460 connected by a spacer sequence of four glycines.

#### RNA isolation and quantitative PCR (qPCR)

RNA was purified using the RNeasy Plus kit (Qiagen, 74134) and cDNA was synthesized using the RNA to cDNA EcoDry kit (Takara), according to the manufacturer’s instructions. Quantitative PCR analysis was performed using Taqman probes from Applied Biosystems for *Atp7a* (Mm00437663), *Atp7b* (Mm00599675) and *Gapdh* (Mm99999915).

#### Enzyme assays and metal measurements

Media harvested from cells after 48 h culture were used to measure lysyl oxidase activity using a LOX activity kit (Abcam, ab112139), according to the manufacturer’s instructions. Metal measurements were performed using inductively coupled plasma mass spectroscopy (ICP-MS), as described previously^52^. GSH and GSSG were measured using the Glutathione Colorimetric Detection Kit (Arbor Assays, K1006-H1), according to the manufacturer’s protocol.

#### Statistical analyses

Statistical analyses were performed using GraphPad Prism 7.0. Values were expressed as mean ± standard error of the mean (SEM) and obtained from at least three biological replicates. Data were analyzed using the standard Student’s *t*-test and were considered significant when p < 0.05. For multiple comparisons, a one-way analysis of variance with post-hoc Tukey test was used. Statistical significance representations: *p < 0.05, **p < 0.01, ***p < 0.001 and ****p < 0.0001.

## Supplementary information

Supplementary information.
