# Supplementary material for: Metallothioneins regulate ATP7A trafficking and control cell viability during copper deficiency and excess
Source: Sci Rep. 2020 May 12;10:7856. doi: 10.1038/s41598-020-64521-3 (PMC7217913; doi:10.1038/s41598-020-64521-3)
Supplement: Supplementary file 1 — Supplementary information. [file 41598_2020_64521_MOESM1_ESM.pdf]

Supplementary Information

Metallothioneins regulate ATP7A trafficking and control cell viability during copper deficiency and excess

Nikita Gudekar[1,2], Vinit Shanbhag[2,3], Yanfang Wang[2,4,6], Martina Ralle[5], Gary A. Weisman[2,3], and Michael J. Petris[1,2,3,4,*]

[1]The Genetics Area Program, University of Missouri, Columbia, MO, 65211; [2]The Christopher S. Bond Life Sciences Center, University of Missouri, Columbia, MO, 65211; [3]The Department of Biochemistry, University of Missouri, Columbia, MO, 65211; [4]The Department of Nutrition and Exercise Physiology, University of Missouri, Columbia, MO, 65211; [5]The Department of Molecular and Medical Genetics, Oregon Health & Science University, Portland, Oregon 97239.

[6]Present address: State Key Laboratory of Animal Nutrition, Institute of Animal Science, Chinese Academy of Agricultural Sciences, Beijing, People's Republic of China, 100193.

Contents include

1) Figure S1
2) Figure S2
3) Figure S3
4) Figure S4
5) Figure S5
6) Figure S6
7) Uncropped western blots

Supplementary Figure 1

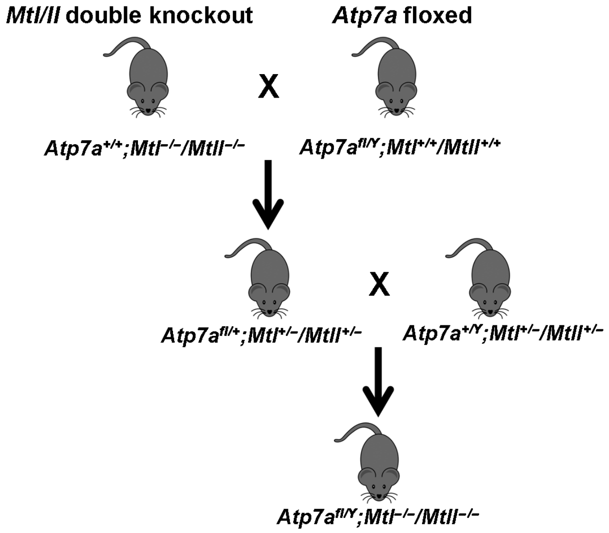

Fig S1: Breeding strategy used to generate *Atp7a* floxed mice carrying deletions in *MtI* and *MtII* genes. The *MtI/II* knockout mice were crossed with *Atp7a* floxed mice to produce F1 offspring that were heterozygous at the *MtI/II* locus and either Wt or floxed at the *Atp7a* locus. These mice were crossed to produce male *Atp7a* floxed mice that were homozygous null for the *MtI/II* alleles.

Supplementary Figure 2

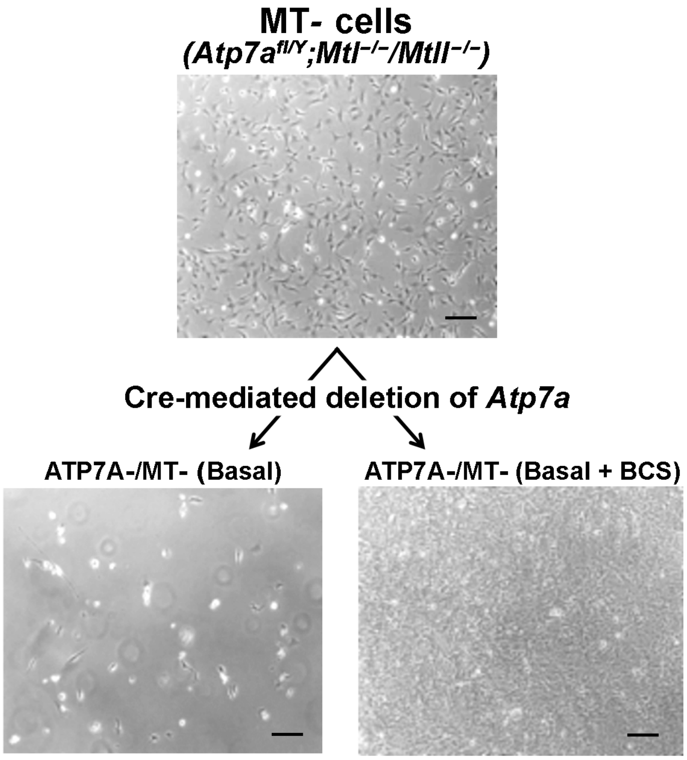

Fig S2: Deletion of the *Atp7a* gene in MT- cells causes a loss of cell viability that is rescued by Cu chelation. MT- cells were seeded overnight in basal medium at approximately 30% confluency (top panel), infected with an adenoviral Cre vector to delete the *Atp7a* gene and then cultured for 5 days in basal media with or without 50 μM BCS (bottom panels). Images were collected by light microscopy; bars, 50 μm.

Supplementary Figure 3

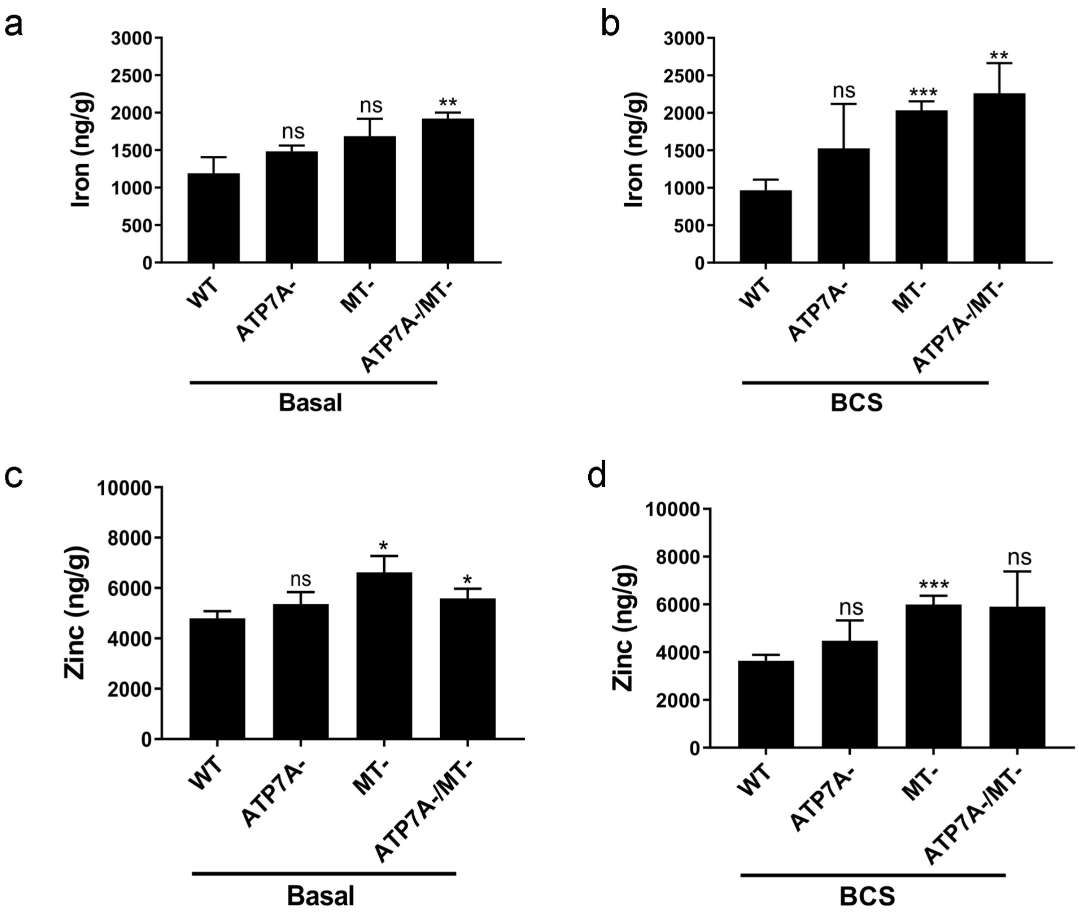

Fig S3: Iron (Fe) and Zinc (Zn) concentrations in each cell line were determined by ICP-MS. Cells were grown for 2 days in medium containing 50 µM of the copper chelator BCS and then exposed for 24 h to either basal media (basal) or media supplemented with 50 µM BCS. Values significantly different from WT are indicated (mean ± SEM; *$p < 0.05$; **$p < 0.01$; ***$p < 0.001$; ns = not significant).

Supplementary Figure 4

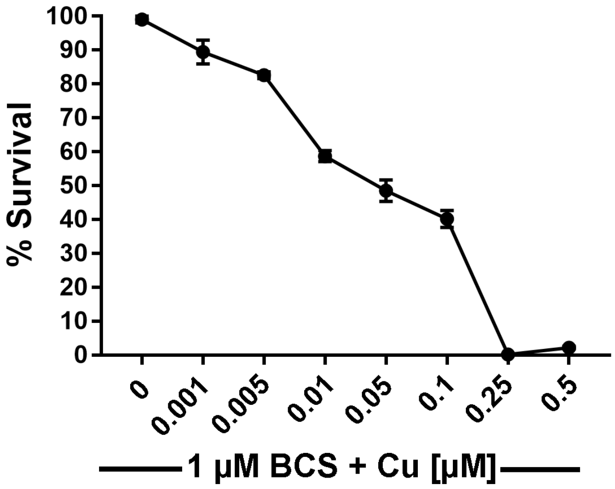

Fig S4: Deletion of ATP7A and MTI/II causes hypersensitivity to sub-micromolar Cu concentrations. ATP7A-/MT- cells were seeded into 6-well dishes (10^3 cells per well) and cultured for 6 days in media supplemented with 1 μM BCS with or without the indicated concentrations of $CuCl_2$. Surviving cells were quantified using the Crystal Violet assay (mean ± SEM).

Supplementary Figure 5

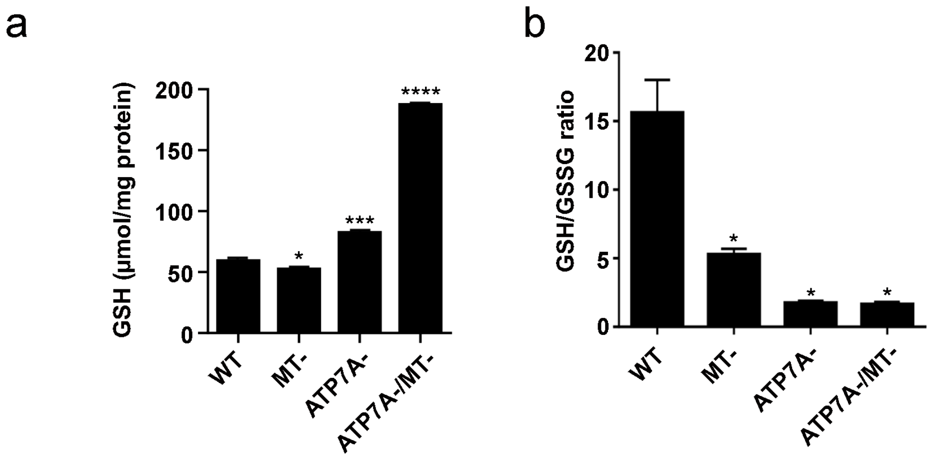

Fig S5: Deletion of ATP7A and MTI/II increases cellular GSH levels and reduces the ratio of reduced glutathione (GSH) to oxidized glutathione (GSSG). Cellular GSH per mg total protein (**a**) and the GSH/GSSG ratio (**b**) were measured in wild type (WT) and mutant cells cultured for 24 h in basal medium. Values are expressed as the mean ± SEM (*p < 0.05; ***p < 0.001; ****p < 0.0001; n = 3).

Supplementary Figure 6

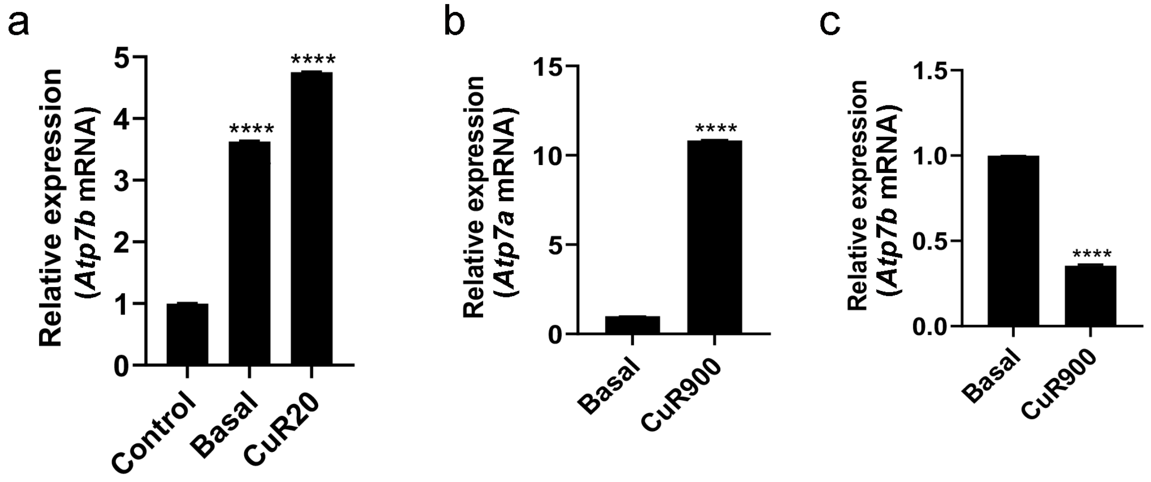

Fig S6: Quantitative RT-PCR analysis of *Atp7a* and *Atp7b* mRNA expression in spontaneously Cu resistant cell lines. (**a**) Parental ATP7A-/MT- (control) cells were selected in either basal medium (basal) or 20 µM $CuCl_2$ (CuR20). Quantitative RT-PCR was used to measure the abundance of *Atp7b* mRNA in each line normalized to *Gapdh* (mean ± SEM; ****p < 0.0001; n = 3). (**b and c**) Parental ATP7A+/MT- cells were grown in either basal medium (basal) or passaged in media containing elevated $CuCl_2$ until resistance to 900 µM was acheived (CuR900). Quantitative RT-PCR was used to measure the abundance of *Atp7a* and *Atp7b* mRNA in each line normalized to *Gapdh* (mean ± SEM; ****p < 0.0001; n = 3).

Full length gels and immunoblot from Figure 1

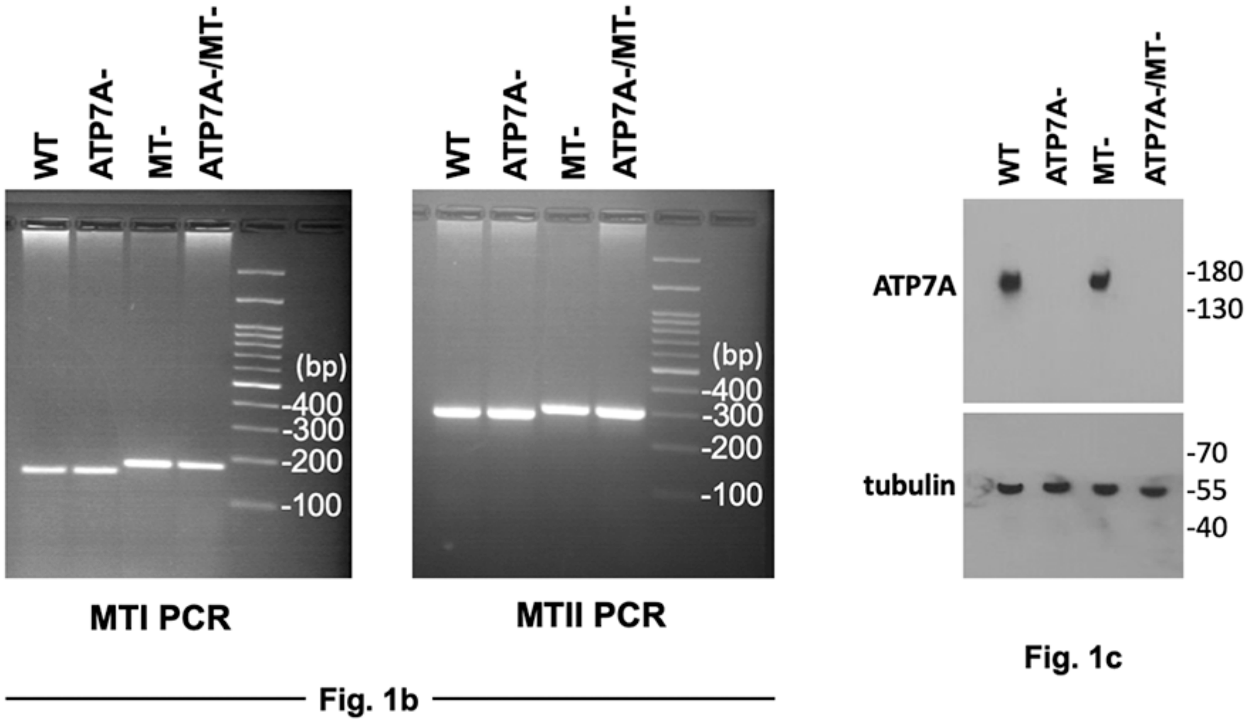

Fig. 1b

Fig. 1c

Full length immunoblots from Figure 4

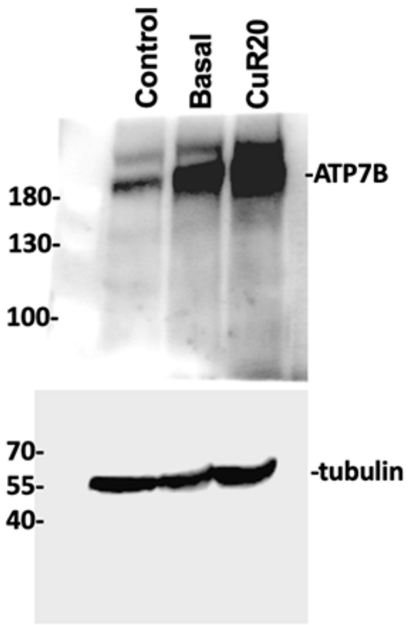

Fig. 4a

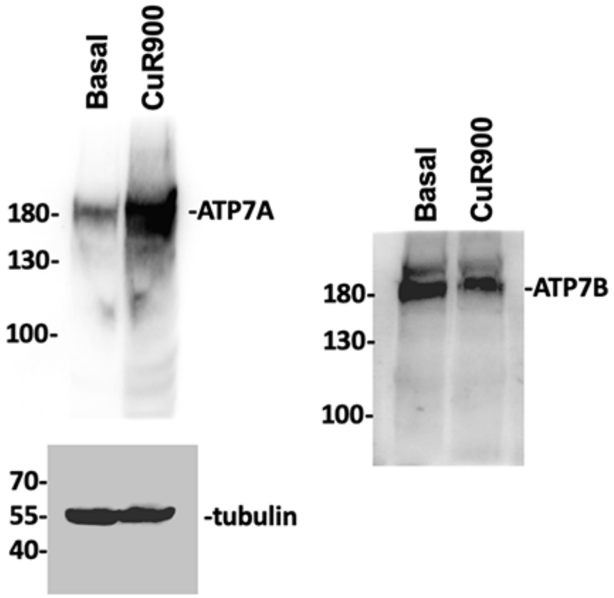

Fig. 4c
